# Genome-wide analysis study of gestational diabetes mellitus and related pathogenic factors in a Chinese Han population

**DOI:** 10.1186/s12884-023-06167-3

**Published:** 2023-12-12

**Authors:** Shufan Yue, Ling Pei, Fenghua Lai, Huangmeng Xiao, Zeting Li, Rui Zeng, Li Chen, Wenzhan Chen, Huiling Liu, Yanbing Li, Haipeng Xiao, Xiaopei Cao

**Affiliations:** Department of Endocrinology, First Affiliated Hospital, Sun Yat-sen University, Guangzhou, 510080 People’s Republic of China

**Keywords:** Gestational Diabetes Mellitus, SNP, GWAS, Susceptibility gene

## Abstract

**Background:**

Gestational diabetes mellitus (GDM) affects the metabolism of both the mother and fetus during and after pregnancy. Genetic factors are important in the pathogenesis of GDM, and associations vary by ethnicity. However, related studies about the relationship between the susceptibility genes and glucose traits remain limited in China. This study aimed to identify genes associated with GDM susceptibility in Chinese Han women and validate those findings using clinical data during pregnancy and postpartum period.

**Methods:**

A genome-wide association study (GWAS) of 398 Chinese Han women (199 each with and without GDM) was conducted and associations between single nucleotide polymorphisms (SNPs) and glucose metabolism were identified by searching public databases. Relationships between filtered differential SNPs and glucose metabolism were verified using clinical data during pregnancy. The GDM group were followed up postpartum to evaluate the progression of glucose metabolism.

**Results:**

We identified five novel SNPs with genome-wide significant associations with GDM: rs62069863 in *TRPV3* gene and rs2232016 in *PRMT6* gene were positive correlated with 1 h plasma glucose (1hPG) and 2 h plasma glucose (2hPG), rs1112718 in *HHEX*/*EXOC6* gene and rs10460009 in *LPIN2* gene were positive associated with fasting plasma glucose, 1hPG and 2hPG, rs927316 in *GLIS3* gene was negative correlated with 2hPG. Of the 166 GDM women followed up postpartum, rs62069863 in *TRPV3* gene was positively associated with fasting insulin, homoeostasis model assessment of insulin resistance.

**Conclusions:**

The variants of rs62069863 in *TRPV3* gene, rs2232016 in *PRMT6* gene, rs1112718 in *HHEX*/*EXOC6* gene, rs927316 in *GLIS3* gene, and rs10460009 in *LPIN2* gene were newly-identified susceptibility loci for GDM in the Chinese Han population. *TRPV3* was associated with worse insulin resistance postpartum.

**Trial registration:**

This study was registered in the Chinese Clinical Trial Registry. Trial registration number: ChiCTR2100043762. Date of first registration: 28/02/2021.

**Supplementary Information:**

The online version contains supplementary material available at 10.1186/s12884-023-06167-3.

## Background

Gestational diabetes mellitus (GDM) is defined as abnormal glucose tolerance that is first detected during pregnancy [1]. It threatens both the mother and offspring during pregnancy and postpartum [2–6]. Previous studies have suggested that women with a history of GDM were at high risk of developing diabetes, dyslipidemia, and cardiovascular events postpartum [4–6]. Further, children whose mothers had GDM, have an increased risk of insulin resistance, obesity, and diabetes [1, 2]. In recent years, the prevalence of GDM in China has undergone a clear increase [7]. Early prediction and treatment could mitigate the maternal and fetal complications associated with GDM [8]. An understanding of the pathogenesis and genetic factors contributing to GDM could facilitate early prediction and treatment of the condition.

To date, epidemiological studies have identified a number of risk factors for GDM, including maternal age, pre-pregnancy body mass index (p-BMI), ethnicity, obesity, family history of type 2 diabetes mellitus (T2DM), and lipid disorders [3, 9]. Additionally, recent studies have also indicated that inflammatory factors, gut microbiome composition, and fatty acid levels were involved in the development of GDM [10–12].

Previous genetic investigations have identified several single nucleotide polymorphisms (SNPs) related to the risk of developing GDM. However, susceptibility varies substantially among women of different ethnicities. With increasing sample sizes and advances in sequencing technology, genome-wide association studies (GWAS) have become the main drivers for a range of discoveries in recent years. Several GWAS analyses have identified SNPs significantly associated with GDM at genome-wide significance, including two genetic variants (rs10830962 in *MTNR1B* gene and rs7754840 in *CDKAL1* gene) identified in a Korean population [13]. In 2013, a GWAS focused on a European population found that *HKDC1* and *BACE2* were associated with GDM [14]. Given the variation in the results of those studies, susceptibility genes are highly likely to vary with ethnicity. However, related studies remain limited in China and the relationship between these genes and glucose traits requires further research. Thus, we conducted this study to search for genes associated with susceptibility to GDM in Chinese Han women and validated our findings using clinical data during pregnancy and postpartum period.

## Methods

### Study population

The study population was conducted in the first affiliated Hospital of Sun Yat-sen University between June and December 2021. The inclusion criteria were as follows: (1) age 18 to 45years; (2) Chinese Han pregnant women; (3) pregnant women received the 75-g oral glucose tolerance test (OGTT). In addition, the exclusion criteria were as follows: (1) diagnosis with diabetes before pregnancy; (2) multiple pregnancy; (3) chronic kidney disease or hepatic dysfunction; (4) a history of organ transplantation, hormonal therapy, or medication with anti-inflammatory agents; (5) malignancy. Diagnosis of GDM was in accordance with the International Association of Diabetes and Pregnancy Study Group criteria [15], based on any of the following after 75-g OGTT: fasting plasma glucose (FPG) level ≥ 5.1 mmol/L and < 7.0 mmol/L, 1 h plasma glucose (1hPG) level ≥ 10.0 mmol/L, or 2 h plasma glucose (2hPG) level ≥ 8.5 mmol/L and < 11.1 mmol/L. The GDM and control groups (pregnant women with normal glucose tolerance) were matched according to age and p-BMI. Control subjects were recruited during the same period as those in the GDM group.

This study was approved by the Medical Ethical Review Committee of the First Affiliated Hospital of Sun Yat-sen University (No. [2017]124 and No. [2021]309) and conducted according to the Declaration of Helsinki. Written informed consent to take part in the study was obtained from all participants. This study was registered in the Chinese Clinical Trial Registry (No. ChiCTR2100043762, first trial registration 28/02/2021).

### Biochemical measurements

Between 24 and 28 weeks of pregnancy, participants were asked to undergo the 75-g OGTT after at least 8 h of fasting. FPG, 1hPG, and 2hPG were performed. Blood glucose and lipid measurements were made using fresh plasma samples. An additional sample was obtained for extraction of total genomic deoxyribonucleic acid (DNA). GDM women received ≥ 30 min of intensive lifestyle education provided by qualified nurses at 1 month after delivery. This education included physical exercise, smoking cessation, limiting alcohol intake, and keeping a healthy diet. In addition, 75-g OGTT were performed at 1–2 years after delivery for GDM women, as the average follow-up time was 17.31 ± 3.44 months. The 33 people who missed the postpartum follow-up include 26 refused postpartum participation (only phone interview) and 7 refused to have blood drawn for further evaluation. FPG, fasting insulin (FINS), 30 min plasma glucose (30minPG), 30 min insulin (30minINS), 2hPG, and 2 h insulin (2hINS) were tested. Glucose levels were determined using a glucose oxidase assay and insulin levels were analyzed by chemiluminescent assays. Blood glucose, insulin and lipids levels were measured using standard enzymatic procedures on an automatic chemistry analyzer (Abbott Aeroset, Chicago, IL, USA). All samples were measured in the laboratory of the Department of Biochemistry of the First Affiliated Hospital of Sun Yat-sen University.

The homoeostasis model assessment of β-cell function (HOMA-β) and insulin resistance (HOMA-IR) were calculated using the following formulae: HOMA-β = 20×FINS (µU/mL)/(FPG [mmol/L] − 3.5[mmol/L]), and HOMA-IR=(FPG [mmol/L] ×FINS [µU/mL]/22.5 [16, 17].

### Genotyping and GWAS analysis

Genomic DNA was extracted using an established genomic DNA kit (Accurate Biotechnology Co., Ltd, Changsha, Hunan, China). The extracted genomic DNA was examined using 1% agarose gel electrophoresis. Each subject was genotyped using an Asian Screening Array bead chip from Illumina (San Diego, CA, USA), following the manufacturer’s protocol. A total of 659,184 SNPs were genotyped for each individual.

A series of quality control criteria were applied. The data exclusion criteria were as follows: (1) per-person missing rate > 5%, (2) per-SNP missing rate > 5%, (3) Hardy–Weinberg disequilibrium *P* value < 1E-6, and (4) minor allele frequency < 5%. Population structure was determined using principal components analysis, and the expected and observed distributions of the *P*-values were compared using Quantile-Quantile plots (Supplementary Figs. 1–2).

### Database analysis

As described in previous studies [18–20], a secondary GWAS signal was identified (*P* < 5 × 10^− 4^) and the top 50 SNPs with the lowest *P* values in this study were selected for analysis. PubMed, GWAS Catalog, and Genotype-Tissue Expression (GTEx) databases were searched to identify SNPs relevant to glucose metabolism up to January 2022. SNPs were converted into genes by searching the NCBI SNP database. For PubMed and GWAS Catalog searches, the keywords were: “gestational diabetes mellitus” or “GDM” or “type 2 diabetes mellitus” or “T2DM” or “insulin” or “glucose”. The Version 7 release of the expression quantitative trait loci (eQTL) summarized data from the GTEx project was used for analysis of the association between SNPs and glucose metabolism-related tissues, including pancreas, liver, small intestine (terminal ileum), adipose (subcutaneous), and muscle (skeletal) [21].

### Statistical analysis

Normally distributed data were expressed as the mean ± standard deviation. Qualitative data were presented as frequencies, and rate or composition ratios. Comparisons between two groups were conducted using the two-tailed unpaired Student’s *t*-test. *P* values < 0.05 were considered statistically significant. Statistical analyses were performed using SPSS 24.0. Associations between SNP genotypes and GDM or glucose traits were analyzed under the additive model using PLINK v1.9 software. Logistic and linear regression modelling were performed assuming an additive genetic model using the PLINK. Linear regression models about SNP genotypes and postpartum glucose traits were adjusted by postpartum obesity. A Manhattan plot of –log_10_ *P* values derived from logistic regression analysis plotted against chromosomal position was generated using the R statistical package. Log_10_ transformation was applied to FPG data, which were non-normally distributed.

## Results

### Baseline characteristics

**Fig. 1:**
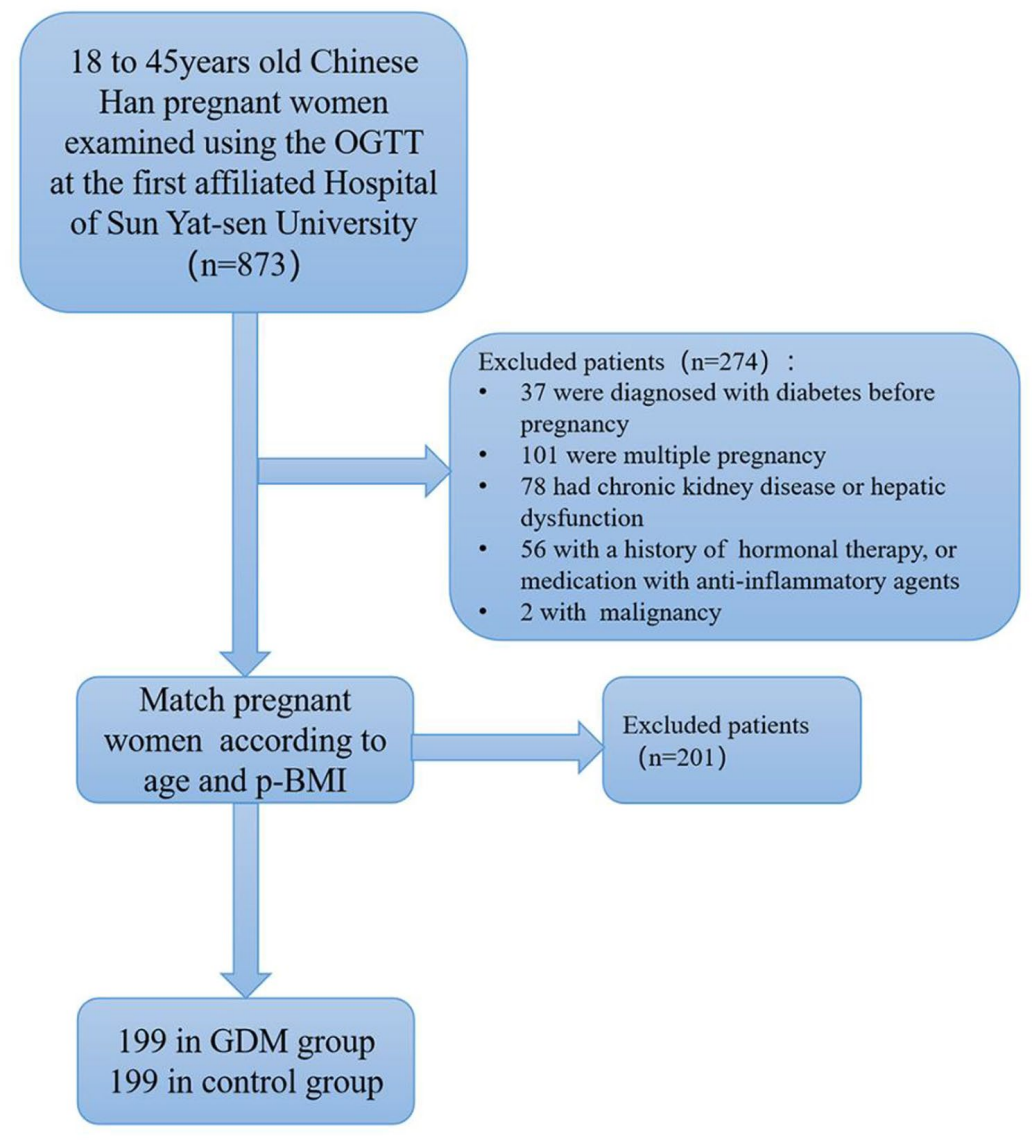
Study flow chart. GDM: Gestational diabetes mellitus; OGTT: oral glucose tolerance test; p-BMI: pre-pregnancy body mass index

The study population comprised 398 participants, including 199 women with GDM and 199 women without GDM (Fig. 1). The native place of the participants was composed of 16 different provinces (Supplementary Table 1). Women in the GDM group aged 32.13 ± 3.65 years, and those in the control group aged 31.57 ± 3.78 years (*P* = 0.135). There were no differences in baseline characteristics between the two groups, as shown in Table 1.

**Table 1:** Baseline characteristics of study participants

	Control group (N = 199)	GDM group (N = 199)	*P* value
Age (years)	31.57 ± 3.78	32.13 ± 3.65	0.135
p-BMI (kg/m^2^)	21.25 ± 2.27	21.53 ± 2.42	0.231
Systolic blood pressure (mmHg)	114.64 ± 8.93	115.99 ± 8.59	0.125
Diastolic blood pressure (mmHg)	70.56 ± 6.21	71.24 ± 6.34	0.281
Heart rate (bpm)	82.77 ± 4.42	83.08 ± 4.80	0.515
Total cholesterol (mmol/L)	6.30 ± 0.99	6.35 ± 1.10	0.581
Triglycerides (mmol/L)	2.12 ± 0.72	2.26 ± 0.97	0.097
High-density lipid cholesterol (mmol/L)	1.97 ± 0.37	1.92 ± 0.36	0.140
Low-density lipid cholesterol (mmol/L)	3.44 ± 0.68	3.52 ± 0.74	0.283

Data were presented as mean values ± standard deviation

GDM: gestational diabetes mellitus; p-BMI: pre-pregnancy body mass index

### GWAS for GDM

**Fig. 2:**
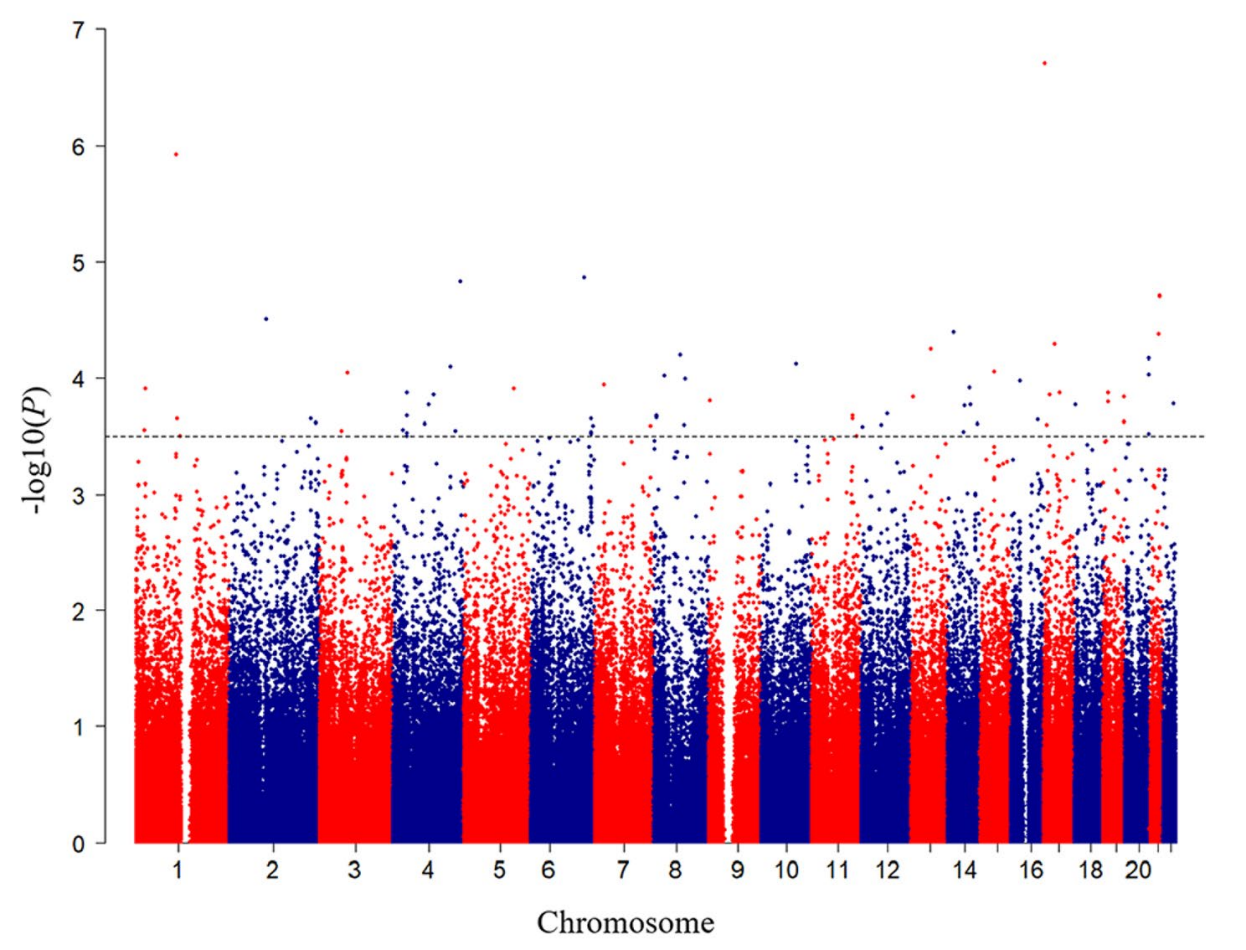
Manhattan plot of genome wide association analysis. Single nucleotide polymorphism locations were plotted on the x-axis according to their chromosomal position. Negative log_10_*P* values derived from logistic regression analysis under an additive model were plotted on the y-axis. The dashed line indicates the threshold of secondary GWAS signal

A total of 301,518 SNPs were retained from all individuals after quality control. A Manhattan plot of the GWAS was presented in Fig. 2. The top 50 SNPs identified by GWAS in increasing order of *P* value were shown in Supplementary Table 2. We converted the top 50 SNPs into genes and 32 genes were screened for relationships with glucose metabolism after searching the PubMed, GWAS catalog, and GTEx eQTL databases. Variants in *TRPV3* gene (rs62069863, *P =* 1.95E-07) and *PRMT6* gene (rs2232016, *P* = 1.20E-06) showed the strongest associations with GDM (Table 2 and Supplementary Table 2). Further, *PRMT6*, *HHEX*, *EXOC6*, *GLIS3*, and *LPIN2* genes were strongly associated with glucose metabolism in at least two databases (Supplementary Fig. 3) and were therefore included in the next stage of the analysis. Although *TRPV3* was only detected as glucose-sensitive in PubMed, it was also included in subsequent analyses, as it exhibited the most significant association with GDM in our population. The associations between these SNPs and GDM were presented in Table 2.

**Table 2:** Associations between SNPs and gestational diabetes mellitus

Gene	SNP	Chromosome	Alleles	Minor allelic frequency		Odds radio (95% confidence interval)	*P* value
				GDM group	Control group		
*TRPV3*	rs62069863	17	**A**/G	0.437	0.263	2.196 (1.629–2.960)	1.95E-07
*PRMT6*	rs2232016	1	**T**/C	0.221	0.095	2.689 (1.785–4.051)	1.20E-06
*HHEX/EXOC6*	rs1112718	10	**A**/G	0.191	0.093	2.289 (1.512–3.507)	8.46E-05
*GLIS3*	rs927316	9	**G**/A	0.151	0.259	0.496 (0.357–0.725)	9.31E-05
*LPIN2*	rs10460009	18	**C**/T	0.562	0.430	2.968 (1.645–5.354)	1.70E-04

Alleles were presented as **minor allele**/major allele

GDM: gestational diabetes mellitus; SNP: single nucleotide polymorphism

### Relationships between identified SNPs and glucose metabolism

To further investigate the roles of these variants in GDM, we first performed an association analysis between the identified SNPs and FPG, 1hPG, and 2hPG during pregnancy. Five SNPs associated with glycemic traits in nongravid populations also demonstrated genome-wide significant associations in pregnant women (Table 3). Specifically, we found positive associations with FPG and SNPs in *HHEX*/*EXOC6* and *LPIN2* gene; 1hPG and SNPs in *TRPV3* gene, *PRMT6* gene, *HHEX*/*EXOC6* gene, and *LPIN2* gene; 2hPG and SNPs in *TRPV3* gene, *PRMT6* gene, *HHEX*/*EXOC6* gene, and *LPIN2* gene. The variant of rs927316 in *GLIS3* gene was significantly negatively correlated with 2hPG. The positive associations between rs62069863 in *TRPV3* gene and both 1hPG and 2hPG reached *P* = 2.60E-04 and *P* = 8.12E-05, with beta values ranging from 0.357 to 0.632 mmol/L and 0.367–0.614 mmol/L per A allele, respectively. The variant of rs2232016 in *PRMT6* gene was strongly positively associated with 1hPG (*P* = 9.12E-06; beta range, 0.593–0.931 mmol/L per T allele) and 2hPG (*P* = 3.28E-04; beta range, 0.417–0.734 mmol/L per T allele). The SNP most strongly positively associated with FPG in *HHEX*/*EXOC6* gene was rs1112718 (*P* = 1.38E-03; beta value range 0.008–0.016 log_10_ (mmol/L) per A allele). Further, rs10460009 in *LPIN2* gene was positive associated with FPG (beta value range, 0.009–0.019 log_10_ (mmol/L) per A allele), while rs10460009 in *LPIN2* gene was positive associated with 1hPG and 2hPG (*P* = 9.48E-03; beta range, 0.394–0.884 mmol/L per A allele; and *P* = 3.13E-04; beta range, 0.595–1.047 mmol/L per A allele, respectively).

**Table 3:** Genome-wide significant associations with glucose metabolism in gravid populations

Gene	SNP	Chromosome	Effect allele in GDM	Effect allele frequency	FPG			1hPG			2hPG		
					Beta	SE	*P* value	Beta	SE	*P* value	Beta	SE	*P* value
*TRPV3*	rs62069863	17	A	0.349	1.07E-03	2.95E-03	7.18E-01	0.490	0.133	2.60E-04	0.491	0.123	8.12E-05
*PRMT6*	rs2232016	1	T	0.158	3.01E-03	3.79E-03	4.27E-01	0.762	0.169	9.12E-06	0.575	0.159	3.28E-04
*HHEX/EXOC6*	rs1112718	10	A	0.142	1.19E-02	4.12E-03	4.04E-03	0.744	0.187	8.10E-05	0.695	0.173	7.34E-05
*GLIS3*	rs927316	9	G	0.205	-4.04E-03	3.56E-03	2.57E-01	-0.256	0.163	1.16E-01	-0.424	0.150	4.96E-03
*LPIN2*	rs10460009	18	C	0.496	1.41E-02	5.35E-03	8.97E-03	0.639	0.245	9.48E-03	0.821	0.226	3.13E-04

GDM, gestational diabetes mellitus; FPG: fasting plasma glucose; SNP: single nucleotide polymorphism; 1hPG: 1 h plasma glucose; 2hPG: 2 h plasma glucose

Further, the relationships between identified SNPs and glucose metabolism during the postpartum period were analyzed. No significant difference was found between the above indices and *PRMT6 or HHEX*/*EXOC6* (Supplementary Table 3), but significant positive relationships were found between *TRPV3* and FINS, 30minINS, HOMA-IR and HOMA-β (Table 4 and Supplementary Table 4). Besides, marginally negative correlation was found between *GLIS3* gene and FPG, while marginally positive correlation was found between *LPIN2* gene and FPG (Supplementary Table 3). The associations between rs62069863 in *TRPV3* gene and FINS reached *P* = 5.31E-05, with beta values ranging from 2.397 to 3.973 µlU/mL per A allele. In addition, strongly positive associations were observed between rs62069863 in *TRPV3* gene and both HOMA-IR and HOMA-β (β = 0.701, *P* = 4.54E-05; β = 45.120, *P* = 2.48E-04).

**Table 4:** The correlation between rs62069863 in *TRPV3* gene and postpartum glucose and insulin levels in women with history of gestational diabetes mellitus

	Beta	SE	*P* value
FPG (mmol/L)	0.075	0.061	2.22E-01
30minPG (mmol/L)	0.311	0.198	1.15E-01
2hPG (mmol/L)	-0.039	0.237	8.69E-01
FINS (µU/mL)	3.185	0.788	5.31E-05
30minINS (µU/mL)	9.092	4.242	3.21E-02
2hINS (µU/mL)	2.140	4.736	6.51E-01
HOMA-IR	0.701	0.172	4.54E-05
HOMA-β	45.120	12.312	2.48E-04
Glycated hemoglobin (%)	-0.012	0.048	7.98E-01

FPG: fasting plasma glucose; FINS: fasting insulin; 2hPG: 2 h plasma glucose; 2hINS: 2 h glucose load plasma insulin;30minINS: 30 min glucose load plasma insulin; 30minPG: 30 min plasma glucose; HOMA-β: homeostatic model assessment for insulin β-cell function; HOMA-IR: homeostatic model assessment for insulin resistance

Adjusted by postpartum obesity

## Discussion

Here we reported the first China-based GWAS of maternal metabolic traits during pregnancy and postpartum. The main findings of this study were that variants of rs62069863 in *TRPV3* gene, rs2232016 in *PRMT6* gene, rs1112718 in *HHEX*/*EXOC6* gene, rs927316 in *GLIS3* gene, and rs10460009 in *LPIN2* gene were associated with GDM and glucose traits. Further, we found that *TRPV3* was positively related with worse insulin resistance of GDM women during the postpartum period.

Six genes at five loci exhibited genome-wide significant associations with maternal metabolic traits, including two genes (*HHEX* and *GLIS3*) that have demonstrated association with GDM in multiple studies [13, 22]. *HHEX* was positively associated with GDM risk in a Korean population and was previously reported as positively associated with T2DM in a Chinese population [23]. *HHEX* participates in pancreas development [24]. Some other studies found that SNPs in *HHEX* were associated with altered β cells secretion [25, 26]. *GLIS3* was associated with GDM in European women [27] and with T2DM in Chinese women. *GLIS3* might control insulin gene transcription and played a role in insulin secretion in β cells [28], while deficiency of *GLIS3* induced apoptosis of the β cells of pancreatic islets and caused diabetes [27, 29, 30]. Hence, our results were consistent with those of previous studies.

We also found evidence for genome-wide significant associations of genes and loci with maternal metabolic traits that have not been previously reported in GDM populations. First, rs62069863 in *TRPV3* gene was the most significant variant with GDM in Chinese Han population in our study and was associated with 1hPG, and 2hPG during pregnancy. Further, our results showed a significant correlation between *TRPV3* and insulin resistance in GDM women after delivery. Cheung et al. reported that activation of *TRPV3* could suppress adipogenesis by inhibiting the insulin receptor substrate 1/phosphoinositide 3-kinase/Akt/forkhead box protein O (FoxO) 1 axis [31], which was an essential glucose metabolism pathway. The biological role of *TRPV3* in glucose and insulin metabolism is unknown. It has been reported that *TRPV* channels can modulate calcium homeostasis and insulin secretion in insulin-producing cells [32–34], which indicated that *TRPV3* may be able to regulate glucose and insulin levels in a similar manner. However, this hypothesis requires further investigations to confirm.

A second novel finding of this study was the association between the rs2232016 T allele of *PRMT6* gene and GDM, 1hPG, and 2hPG during pregnancy. The SNP rs2232016 is located in the only exon of *PRMT6* gene and plays an important role in regulation of *PRMT6* expression. It is highly likely that *PRMT6* participates in glucose metabolism. Arginine methylation by *PRMT6* was critical in maintaining adenosine cyclophosphate response element binding protein-regulated transcriptional coactivator 2 interaction on gluconeogenic promoters, and knockdown of hepatic *PRMT6* in mice would lead to FPG decreasing [35]. Furthermore, Choi et al. reported that *PRMT1* regulated FoxO3 through modulation of *PRMT6* [36]. The absence of *PRMT1* in skeletal muscle increased *PRMT6* specifically methylates FoxO3, leading to enhanced expression of the autophagic marker. In addition, according to GTEx database, significant differences in the expression levels of the three genotypes of rs2232016 in *PRMT6* gene were found in liver. It was reported that *PRMT6* could regulated lipid transport [37]. Thereby, rs2232016 in *PRMT6* gene might affect hepatic lipid metabolism. However, these issues still require further explorations.

In addition, we reported the first association between rs1112718 in *HHEX*/*EXOC6* gene and GDM, although this SNP was previously reported as a T2DM susceptibility locus [38]. *EXOC6* was associated with guanosine triphosphate binding protein-10 in adipocytes and participated in insulin signaling by influencing glucose transporter 4 (GLUT4) translocation [39]. GLUT4 controlled glucose transport into fat and muscle tissue in response to insulin and also into muscle during exercise [40]. However, rs1112718 was located in an intergenic region between *HHEX* and *EXOC6*, and its function remained unclear. Another locus, rs10460009 in *LPIN2* gene, which was associated with FPG, 1hPG, and 2hPG in the current study, has not been previously associated with GDM risk or metabolic traits in humans. Aulchenko et al. found that *LPIN2* was associated with T2DM and fat distribution [41], but studies about the role of LPIN2 in glucose metabolism are still limited.

Although the distributions of five new SNPs in Chinese Han population of our study were consistent with other population worldwide [42–44], the relationship between these five SNPs and GDM was not found in previous studies. There were two GWAS studies in other ethnicities, in Korea [13] and in European-American [14]. The genes found in this study were different from those two studies. However, those two studies reported different genes to each other. This revealed the genetic basis of GDM in different ethnicities. Therefore, it is necessary for different countries and regions to seek their own susceptibility genes.

This study demonstrated that five novel SNPs with genome-wide significant associations with GDM could contribute to the early prediction of GDM. The findings may provide new knowledge to our understanding of the causes of GDM. There are several limitations in our study. First, due to the relatively small sample size and slight lack of representative for Chinese Han population, our results require more Han women with GDM to expand representativeness and further verification by database mining and analysis of clinical factors. Moreover, this study did not include analysis of glycemic changes in offspring. A large-scale multicenter study with long-term follow-up should be performed in the future to validate our findings and expand representativeness. In addition, animal experiments are needed to further confirm the results of our study. Thus, further experiments are required to verify these findings.

## Conclusions

In summary, our study was the first to show that rs62069863 in *TRPV3* gene, rs2232016 in *PRMT6* gene, rs1112718 in *HHEX*/*EXOC6* gene, rs10460009 in *LPIN2* gene and rs927316 in *GLIS3* gene were susceptibility loci for GDM and were all significantly associated with blood glucose levels during pregnancy. Further, our data indicated that *TRPV3* might participate in insulin resistance in GDM women postpartum.

### Electronic supplementary material

Below is the link to the electronic supplementary material.

Supplementary Material 1

Supplementary Material 2

## Data Availability

The datasets used and/or analyzed during the current study are available from the corresponding author on reasonable request.

## Abbreviations

DNA: Deoxyribonucleic acid
eQTL: Expression quantitative trait loci
FINS: Fasting insulin
FoxO: Forkhead box protein O
FPG: Fasting plasma glucose
GDM: Gestational diabetes mellitus
GLUT4: Glucose transporter 4
GTEx: Genotype-Tissue Expression
GWAS: Genome-wide association study
HOMA-β: Homoeostasis model assessment of β-cell function
HOMA-IR: Homoeostasis model assessment of insulin resistance
OGTT: Oral glucose tolerance test
1hPG: 1 h plasma glucose
p-BMI: Pre-pregnancy body mass index
SNP: Single-nucleotide polymorphism
2hINS: 2 h glucose load plasma insulin
2hPG: 2 h plasma glucose
T2DM: Type 2 diabetes mellitus
30minINS: 30 min glucose load plasma insulin
30minPG: 30 min plasma glucose
