# Supplementary material for: Genome-wide analysis study of gestational diabetes mellitus and related pathogenic factors in a Chinese Han population
Source: BMC Pregnancy Childbirth. 2023 Dec 12;23:856. doi: 10.1186/s12884-023-06167-3 (PMC10714520; doi:10.1186/s12884-023-06167-3)
Supplement: Supplementary file 1 — Supplementary Material 1 [file 12884_2023_6167_MOESM1_ESM.docx]

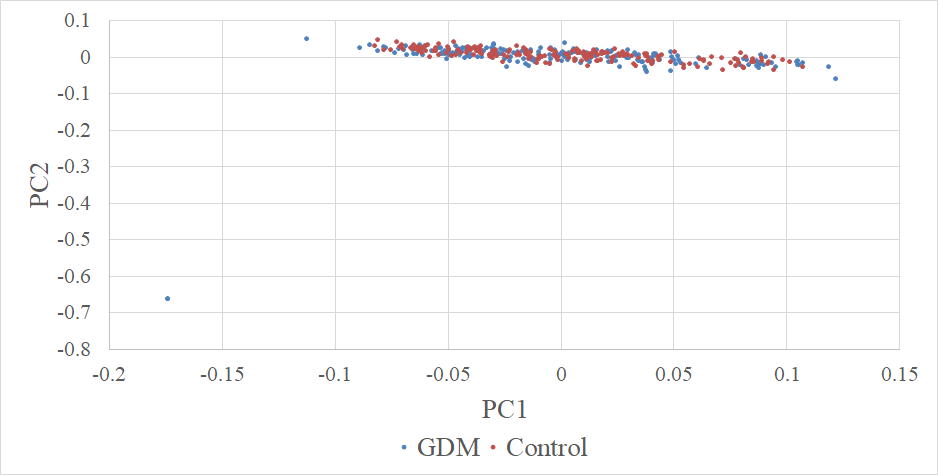


**Supplementary Fig. 1** Principal component analysis (PCA) between GDM and control groups

The clinical variables was whether or not GDM (199 with GDM in GDM group, and 199 without GDM in control group). The first 10 PCA factors were retained. PCA was performed using PLINK v1.9 software.


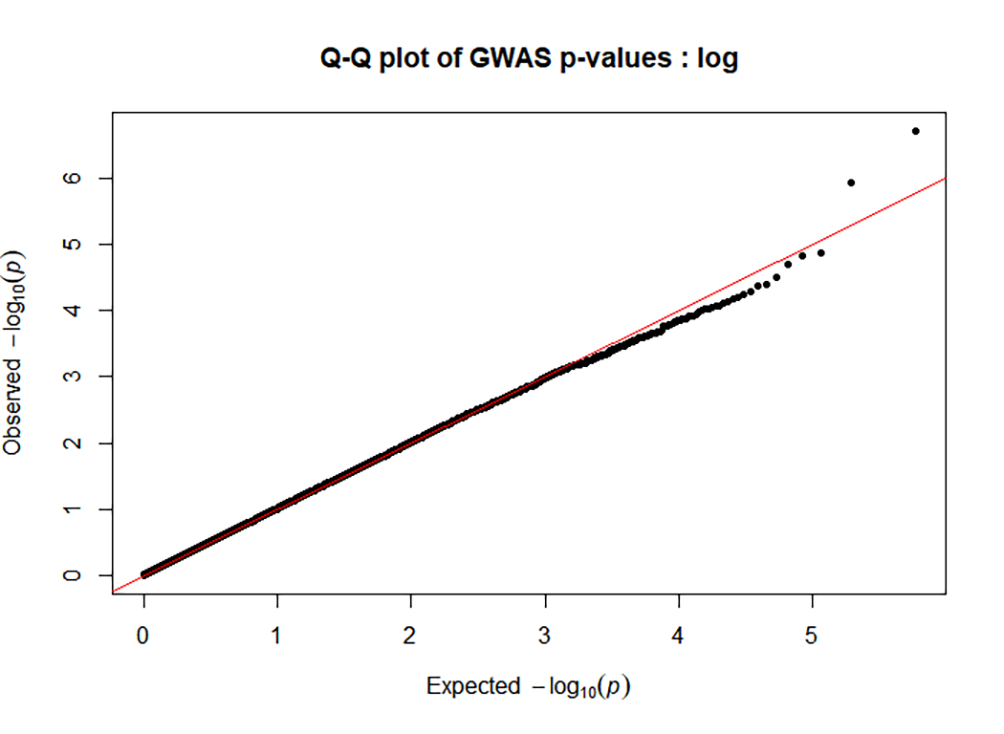


**Supplementary Fig. 2** Quantile-quantile (QQ) plot of genome wide association analysis


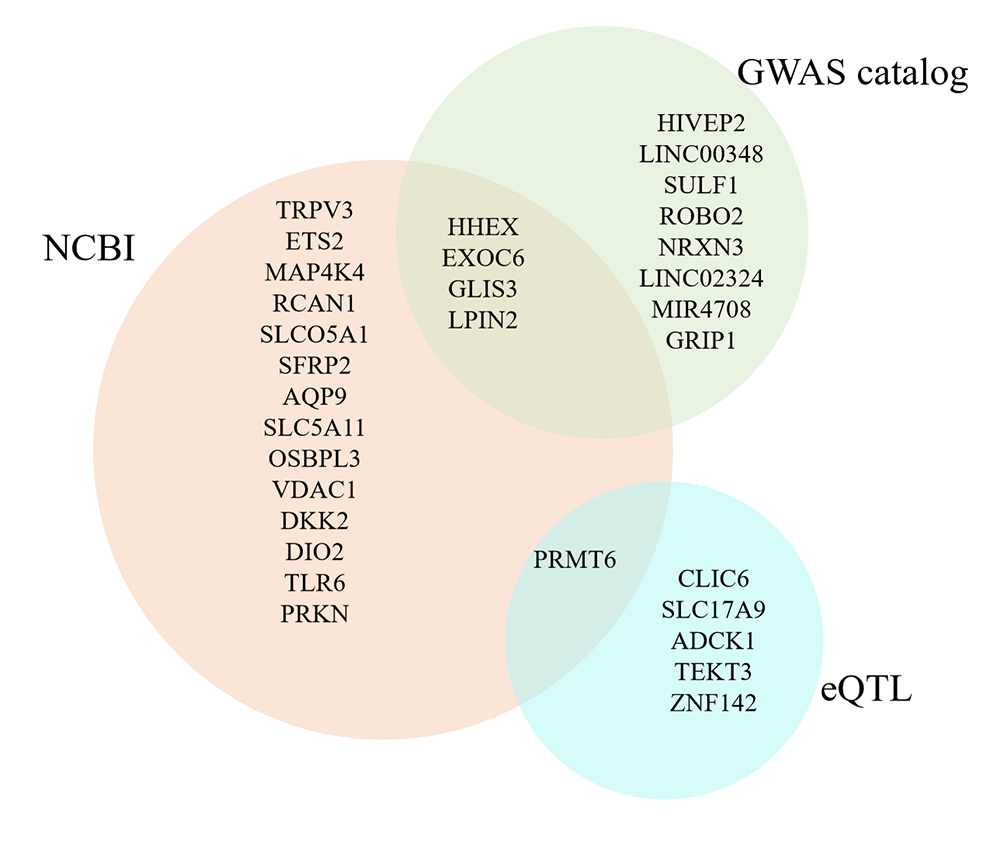


**Supplementary Fig. 3** Genes associated with glucose metabolism in PubMed, GWAS catalog, and GTEx eQTL databases
